# Therapeutic Duration and Extent Affect the Effect of Moxibustion on Depression-Like Behaviour in Rats via Regulating the Brain Tryptophan Transport and Metabolism

**DOI:** 10.1155/2019/7592124

**Published:** 2019-08-27

**Authors:** Hao Li, Lan Sang, Xing Xia, Ruirui Zhao, Mingyue Wang, Xiaofei Hou, Jiawei Xiong, Tiemin Cao, Xiaoquan Liu, Jianbin Zhang

**Affiliations:** ^1^Acupuncture and Moxibustion Department, Jiangsu Provincial Second Chinese Medicine Hospital/The Second Affiliated Hospital of Nanjing University of Chinese Medicine, Nanjing, 210017, China; ^2^Center of Drug Metabolism and Pharmacokinetics, China Pharmaceutical University, Nanjing, 210009, China

## Abstract

Moxibustion has been widely accepted as an alternative therapy for major depressive disease (MDD). However, the efficacy of moxibustion treatment on MDD is highly variable because of its irregular operation. This study was designed to investigate how therapeutic duration and extent influence the anti-depression effect of moxibustion and the underlying mechanism involved. Rats with lipopolysaccharide-induced depression-like behavior were treated by moxibustion treatment. The anti-depression effect was determined by forced swimming test and open field test. Tryptophan (Trp) transport and its metabolism to serotonin (5-HT) and kynurenine (Kyn) were evaluated to explore the anti-depression mechanism. The results showed that moxibustion treatment could alleviate the depression-like behavior in rats. Trp transport and 5-HT generation were significantly increased, and the Trp-Kyn pathway was moderately inhibited by moxibustion. Prolonged therapy could be beneficial to the anti-depression effect by promoting the brain uptake of Trp and shifting the Trp metabolism to 5-HT. An enhanced therapeutic extent could increase 5-HT generation. In conclusion, this study determined that the anti-depression effect of moxibustion involves improved Trp transport and metabolism. The therapeutic duration benefits antidepressant effects, but the complex influence of the therapeutic extent on moxibustion efficacy requires further studies.

## 1. Introduction

Moxibustion is a traditional Chinese medicine that uses an ignited moxa giving off heat at an acupoint on the body surface. Several studies showed the efficacy of moxibustion combined with other antidepressants [1–3]. For instance, the combination of moxibustion, acupuncture, and selective serotonin reuptake inhibitors (SSRIs) demonstrated superior efficacy on major depressive disorder (MDD) patients compared with sham acupuncture–SSRI therapy [4]. However, empirically designed moxibustion strategies make the efficacy of moxibustion on MDD highly variable. The efficacy of moxibustion is influenced by multiple factors in moxibustion treatment, such as therapeutic duration, extent, and frequency. To achieve optimal and stable efficacy, understanding the underlying mechanism involved and how these factors affect the efficacy of moxibustion treatment on MDD is needed [5].

**Figure 1:**
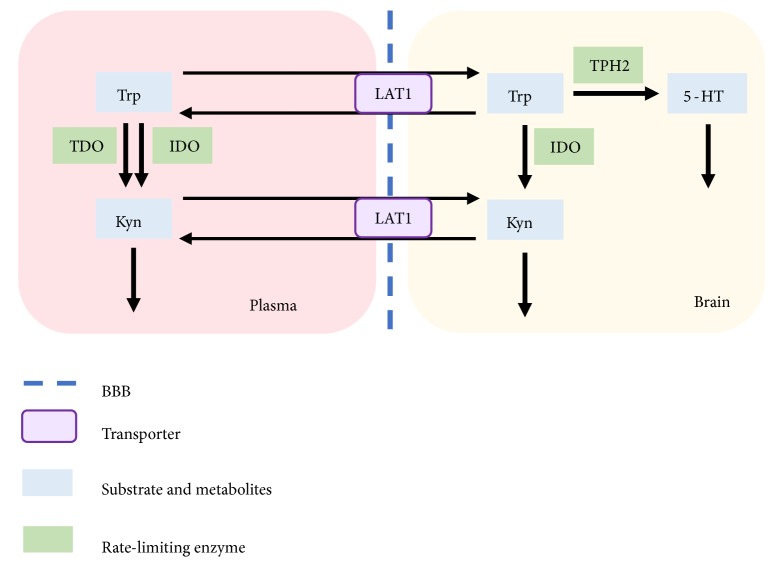
*Metabolism pathways of Trp in human plasma and brain.* TRP: tryptophan, the precursor of kynurenine and serotonin; KYN: kynurenine; 5-HT: serotonin; TPH2: tryptophan hydroxylase, key enzyme of Trp-5-HT pathway; TDO: tryptophan 2,3-dioxygenase, key enzyme of Trp-Kyn pathway in liver; IDO: indoleamine 2,3- dioxygenase, key enzyme of Trp-Kyn pathway in tissues except liver; LAT1: large neutral amino acid transporter, transporter for Trp and Kyn on Blood-Brain-Barrier.

According to the serotonin–kynurenine hypothesis of depression, tryptophan (Trp) deficiency and the imbalanced metabolism of Trp in the brain are largely related to MDD [6–8]. As shown in Figure 1, Trp is the precursor of both serotonin (5-HT) and kynurenine (Kyn). The transporter-mediated uptake of Trp, an essential amino acid, from plasma is a determinant of the available brain Trp. A reduced plasma Trp level is always accompanied by insufficient brain Trp availability and serotonin synthesis [9–11]. The Trp-Kyn and Trp-5-HT pathways are the major Trp metabolism pathways related to depression. In humans, 5% of Trp is metabolized via the serotonin pathway by tryptophan hydroxylase (TPH2). The majority (about 90%–95%) of Trp is converted by indoleamine 2,3-dioxygenase and tryptophan 2,3-dioxygenase into Kyn [12]. The over-activation of the Trp-Kyn pathway, for instance, by inflammation potentially promotes the development of depression through competition of Trp with the Trp-5-HT pathways or the generation of neurotoxic compounds.

Thermal stimulation is the most acceptable mechanism of action in moxibustion treatment [13]. The therapeutic benefit of thermal stimulation on MDD has been demonstrated in preclinical and clinical studies [14, 15]. Thermal therapy was reported to change physiological properties, such as blood perfusion [16] and enzyme activity [17]. Brain Trp transport and metabolism are related to brain blood perfusion and metabolic enzyme activity, which might be affected by thermal stimulation. Thus, we hypothesize that the antidepressant-like behavior resulting from moxibustion might be related to the altered Trp transport and metabolism in the brain. As the changes in physiological properties by thermal stimulation are both duration and extent dependent, the therapeutic duration and extent of moxibustion are considered as two pivotal factors that determine its efficacy on depressant-like behavior. To test this hypothesis, we treated rats with depression-like behavior with moxibustion under different durations and extents, determined the rats' antidepressant-like behavioral response based on forced swimming test (FST) and open field test (OFT), and quantified the plasma and brain Trp, 5-HT, and Kyn levels.

## 2. Materials and Methods

### 2.1. Animals

Wistar rats (N=77; 180–200g, 8 weeks old) were obtained from Shanghai SIPPR*-*Bk. The rats were kept in an air-conditioned room with a 12 h light/dark cycle with free access to food and water. During the experiment, the rats were not restrained in activity, and they had free access to food and water, except during moxibustion treatment and the behavioral tests. The experiment procedures were approved by the Animal Care and Use Committee at China Pharmaceutical University.

### 2.2. Animal Handing

After a two-week adaptive breeding, the rats (N=77; 220–240 g, 10 weeks old) were randomly divided into seven groups, as shown in Table 1. A depressive rat model was induced by 0.5 mg/kg intraperitoneal (*i.p.*) injection of freshly prepared lipopolysaccharide (LPS, Escherichia coli 055:B5) in sterile endotoxin-free saline. A single moxibustion treatment was carried out immediately after LPS injection. A smoke-free cylinder-shaped moxa (Nanyang Aixin Moxa Biological Products Co., Ltd., Nanyang, China) was used. The moxa floss was seven years old and was carbonized before it was made into moxa sticks. The dimension of the moxa is 14 mm in diameter and 27 mm in height. During moxibustion treatment, the rats were maintained within a fixation device. A moxa was fixed above the device with a flexible copper wire. The moxa was adjusted to aim Dazhui (GV14). Ignition was started at the bottom end of the moxa. To avoid distance change after burning, moxa ash was cleared every 5 min. The rats were treated with moxibustion under different durations and extents. The duration of moxibustion treatment was manually measured. The temperature of the rat skin surface under different moxibustion strategies was measured with an infrared thermometer (testo 830-T4, Testo SE & Co. KGaA, Germany). The extent of moxibustion treatment was controlled by the distance between the moxa and the acupoint (no treatment: 25°C, 7.5 cm: 28°C, 5.0 cm: 33°C, 2.5 cm: 37°C). During the treatment, the distance was strictly measured and controlled. Measurement began when a rat was fixed under an ignited moxa, and the rat was softly moved away when the time was up.

Plasma samples were collected 0.5 h before and 2, 4, 6, 8, 10, and 24 h after LPS injection. Then, 200 *μ*L of blood collected from the orbital plexus was centrifuged at 4,000 rpm for 10 min before the supernatant was transferred into a centrifuge tube and stored at −80°C until analysis. In each group, the rats were sacrificed at 4 h (n = 3) and 8 h (n = 3) after LPS injection to collect the brain samples. In addition, five rats were used for depression-like behavioral test at 23 h (n = 5) and then sacrificed to collect the brain sample at 24 h post-LPS injection. Brain hippocampus was dissected on ice and stored at −80°C until analysis.

### 2.3. Depression-Like Behavioral Test

#### 2.3.1. Open Field Test (OFT)

The OFT was carried out at 23 h post-LPS injection. The apparatus consisted of a square arena (100 cm × 100 cm) with 50 cm high walls. To start each test session, a single rat was gently placed in a particular corner of the arena and allowed to explore the arena for 5 min. The sessions were recorded by a video camera placed above the arena. The total ambulatory distance was measured by ANY-maze™ Video Tracking System (Stoelting Co. Wood Dale, IL, USA).

#### 2.3.2. Forced Swimming Test (FST)

After a short rest, the FST was performed. The apparatus was a transparent cylindrical glass container measuring 50 cm in height and 18 cm in diameter. The rats were forced to swim for 6 min, and the immobility time during the last 5 min was manually measured by two observers who were blinded to the experiment. The rats were considered immobile when they ceased struggling, remained floating motionless, and only made movements that were necessary to keep their heads above the water.

#### 2.3.3. Behavioral Score Calculation

MDD is a comprehensive and complicated disease. A self-rating depression scale (SDS) was designed to assess the level of depression of MDD patients. An SDS includes up to 20 clauses to assess the level of depression comprehensively. Similar to an SDS, different behavioral tests can reveal the behavioral characteristics of depressed or moxibustion-treated rats in different aspects. The FST was considered to reflect a state of lowered mood or hopelessness in depressive rats [18], whereas the OFT reflects sickness [19, 20]. To assess the level of depression comprehensively in preclinical studies, we scored the rats according to the FST and the OFT. The grading criteria are as follows: first, the results of normal rats were artificially set as 100 points; second, the percentage of deviation from normal rats was defined as the lost points for the model or the mox-treated rats.

### 2.4. Trp, Kyn and 5-HT Determination

#### 2.4.1. Plasma Trp and Kyn Levels Determination

Pseudoephedrine (PE) was used as internal standard for Trp and Kyn detection in plasma samples by LC-MS/MS analysis. 50 *μ*L plasma sample was diluted with 50 *μ*L water (containing 2 *μ*g/mL PE). Protein was precipitated with 100 *μ*L perchloric acid (10% in water, v/v). After centrifugation at 15,000 rpm for 10 min, an 80 *μ*L aliquot was transferred to the vial and 5 *μ*L was injected for analysis. The plasma samples were injected into a Hanbon Hedera ODS-2 column (150 mm × 2.1 mm; 5 *μ*m) and analyzed on a Shimadzu HPLC system (Shimadzu Corporation, Kyoto, Japan) coupled with a TSQ Quantum Access mass spectrometer (Thermo Fisher). The mobile phase was composed of solvent A (ultrapure water, 84%) and solvent B (acetonitrile, 16%). The flow rate was 0.2 mL/min. Mass spectrometry parameters were set as follows: ion spray voltage: 5.0 kV (+); sheath gas pressure: 55 Arb; Aux gas pressure: 5 Arb; capillary temperature: 350°C. Multiple reaction monitoring (MRM) transitions and individual parameters applied for plasma analytes are summarized in Table 2.

#### 2.4.2. Brain Trp, 5-HT and Kyn Levels Determination

Caffeic acid (CA) was selected as internal standard for Trp, Kyn and 5-HT detectation in hippocampus samples according to previous reports [21, 22]. 100 *μ*L of potassium phosphate buffer (50mM, pH 6.0) containing 40 mM ascorbic acid was added to 50 mg of rat hippocampus tissue, and the mixture was homogenized in an ice bath followed by centrifugation at 15,000 rpm for 10 min. 50 *μ*L supernatant was extracted and added an aliquot of 150 *μ*L of ice-cold acetonitrile. the mixture was vortexed for 3 min followed by centrifugation at 15,000 rpm for 10 min. The supernatant (150 *μ*L) was dried under vacuum at 25°C room temperature (about 25°C). For derivatization, 50 *μ*L of borate buffer (sodium teraborate, 100 mM in water) and 50 *μ*L of benzoyl chloride (2.0% in acetonitrile, v/v) were added to the residue and vortexed under room temperature for 5 min. After centrifugation at 15,000 rpm for 10 min, an 80 *μ*L aliquot was transferred to the vial and 20 *μ*L was injected for analysis.

The hippocampus samples were injected into a Hanbon Hedera ODS-2 column (150 mm × 2.1 mm; 5 *μ*m) and analyzed on a Shimadzu HPLC system (Shimadzu Corporation, Kyoto, Japan) coupled with a TSQ Quantum Access mass spectrometer (Thermo Fisher). The mobile phase was composed of solvent A (0.1% formic acid and 2.0 mM ammonium acetate in water) and solvent B (acetonitrile). The solvent gradient used was as follows: 30% B from 0 to 1.5 min, increased to 65% B at 3.5 min and held for 1.5 min, increased to 75% B at 8.0 min, and then decreased to 30% at 10.0 min followed by 3.0 min for equilibration. The flow rate was 0.2 mL/min. Mass spectrometry parameters were set as follows: ion spray voltage: 5.0 kV (+); sheath gas pressure: 20 Arb; Aux gas pressure: 5 Arb; capillary temperature: 350°C. Multiple reaction monitoring (MRM) transitions and individual parameters applied for hippocampus analytes are summarized in Table 3.

### 2.5. Data and Statistical Analyses

The transport capacity of Trp in brain blood vessels was quantified by comparing the Trp exposure in plasma and the hippocampus. Trp exposure was determined by the area under the drug concentration curve (AUC), which was calculated with the linear trapezoidal rule. The metabolism activation of the Trp-5-HT and Trp-Kyn pathways was evaluated by determining the AUC ratios of brain Trp and 5-HT (or Kyn) [23, 24].

In the correlation analysis, data from individual rats were utilized to explore the relationship between the Trp transport/metabolism indexes and the depression-like behavioral score. The AUC of Trp, 5-HT, and Kyn in the hippocampus was not calculated, as only one sample could be collected for each rat. Thus, the average concentrations of Trp, 5-HT, and Kyn in the hippocampus were used to determine the Trp transport and activity of the Trp-5-HT and Trp-Kyn metabolism pathways.

In this study, the data were presented as means ± S.E.M. Before the comparisons, the normality of residuals was analyzed to determine the homogeneity of variance. Student t test was used for a single comparison of the control group versus the model group. One-way ANOVA was used for the comparison of the model group versus the moxibustion-treated groups, followed by the least significant difference post hoc test. P < 0.05 was the accepted level of significance.

## 3. Results

### 3.1. Effect of Moxibustion Treatment on Depression-Like Behaviors

**Figure 2:**
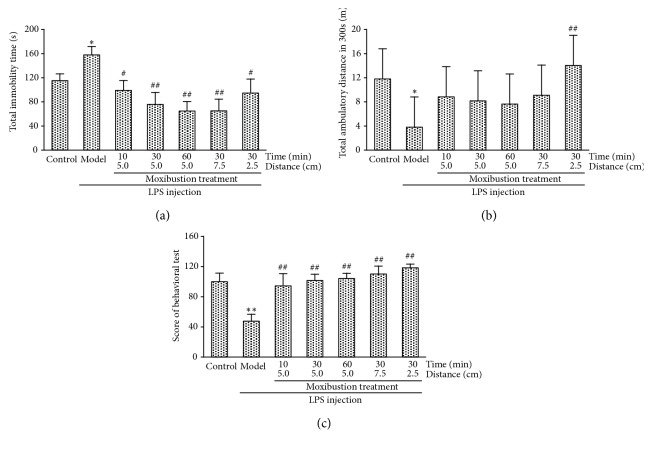
*Depression-like behavioral test results.* (a) Effect of moxibustion treatment on rat immobility time in water, (b) Effect of moxibustion treatment on locomotor activity, (c) Effect of moxibustion treatment on depression-like behavioral scores of depressive-behavior rats. (*∗* P < 0.05, *∗∗* P < 0.01: compared with Control group; # P < 0.05, ## P < 0.01: compared with Model group).

As shown in Figure 2(a), the rats with LPS-induced depression-like behavior spent a much longer immobility time in the FST compared with the control group (P < 0.05). Moxibustion treatment significantly reduced the immobility time compared with the case of the model group (F[5, 24] = 3.675, P < 0.05), suggesting that the lowered mood and hopelessness could be improved by moxibustion treatment. Moreover, the immobility time declined with the prolonged moxibustion treatment duration, but the enhanced extent exerted a suboptimal benefit.

As shown in Figure 2(b), LPS injection significantly decreased the total ambulatory time in the OFT (P < 0.05). Moxibustion treatment with the highest thermal effect significantly increased the travelling distance compared with the case of the LPS group (P < 0.05). Despite the lack of a significant difference, the travelling distance showed a declining trend with the prolonged treatment duration. These results implied that the efficacy of moxibustion in sickness is highly related to therapeutic extent rather than to duration.

To assess the level of depression comprehensively in this study, we scored the rats according to the FST and OFT results. As shown in Figure 2(c), the LPS rats showed a significant decrease in behavioral score compared with the control group (P < 0.01). Moxibustion treatment effectively reversed the alteration (F[5,24] = 6.322, P < 0.01). Therapeutic extent appeared more sensitive than duration was in improving the behavioral score.

### 3.2. Effect of Moxibustion Treatment on Trp Transport and Metabolism

#### 3.2.1. Effects of Moxibustion Treatment on Trp and Kyn Levels in Plasma

**Figure 3:**
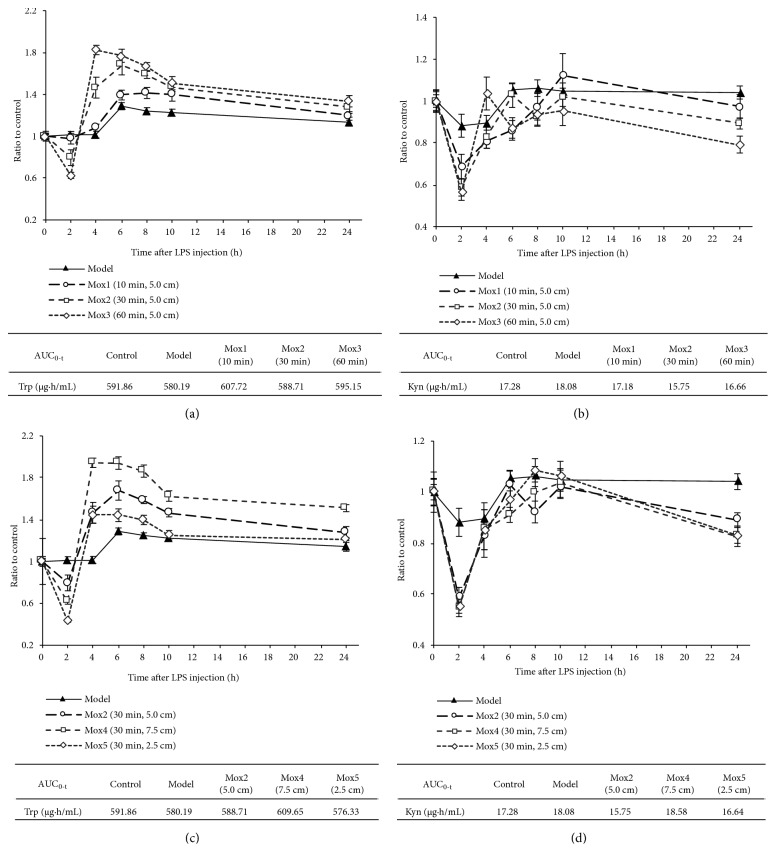
*Effects of moxibustion treatment on Trp and Kyn levels in plasma.* (a) effect of moxibustion treatment duration on plasma Trp levels, (b) effect of moxibustion treatment duration on plasma Kyn levels, (c) effect of thermal effect extent on plasma Trp levels, (d) effect of thermal effect extent on plasma Kyn levels.

LPS injection produced a slight increase in plasma Trp and Kyn levels (Figure 3). The plasma Trp levels (Figure 3(a)) were further elevated, whereas the Kyn levels (Figure 3(b)) were reduced by moxibustion treatment with therapeutic duration-dependent profiles. The therapeutic extent is inversely related to the plasma Trp level elevation (Figure 3(c)). No influence of therapeutic extent on plasma Kyn levels was observed in our analysis (Figure 3(d)).

#### 3.2.2. Effects of Moxibustion Treatment on Trp Uptake in the Hippocampus

**Figure 4:**
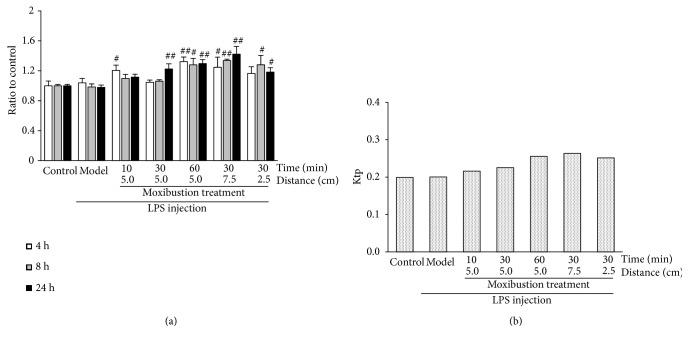
*Effects of moxibustion treatment on Trp uptake in hippocampus.* (a) Effect of moxibustion treatment on Trp content in hippocampus, (b) Effect of moxibustion treatment on the partition coefficient of Trp between hippocampus and plasma (Ktp). (# P < 0.05, ## P < 0.01: Compared with Model group).

As shown in Figure 4(a), a significant influence was observed in hippocampus Trp content with moxibustion but not with LPS treatment. Hippocampus Trp is transported from plasma. Plasma Trp level and transport capacity are two determinants of hippocampus Trp exposure. Thus, the partition coefficient of Trp (Ktp) between the hippocampus and plasma was used to evaluate transport capacity. As shown in Figure 4(b), a prolonged treatment duration increased the Trp transport capacity in the hippocampus, with the Ktp increasing from 0.22 (10 min) to 0.23 (30 min) and to 0.26 (60 min). The enhanced treatment extent exerted a nonlinear influence on Trp transport property, in which Ktp was altered from 0.26 (7.5 cm) to 0.23 (5 cm) and to 0.25 (2.5 cm).

#### 3.2.3. Effect of Moxibustion Treatment on the Trp-5-HT Pathway in the Hippocampus

**Figure 5:**
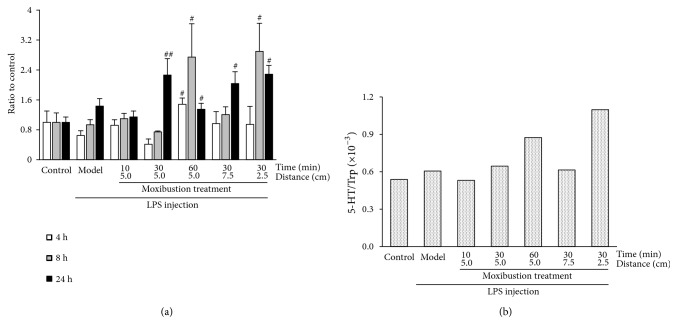
*Effects of moxibustion treatment on Trp metabolism via 5-HT in hippocampus.* (a) Effect of moxibustion treatment on 5-HT content in hippocampus, (b) Effect of moxibustion treatment on the ratio of hippocampus exposes of 5-HT to Trp (5-HT/Trp). (# P < 0.05, ## P < 0.01: Compared with Model group).

A fast decrease and slow recovery in hippocampus 5-HT content was observed at 4 h, 8 h, and 24 h after LPS injection (Figure 5(a)). Hippocampus 5-HT is solely generated by Trp metabolism. To evaluate the metabolism capacity of Trp to 5-HT, the ratio of hippocampus exposure of 5-HT to Trp (5-HT/Trp) was calculated. As shown in Figure 5(b), the 5-HT/Trp metabolism capacity increased with both the duration (0.53×10^−3^, 0.65×10^−3^, and 0.87×10^−3^) and temperature (0.61×10^−3^, 0.65×10^−3^, 1.10×10^−3^) of moxibustion treatment.

#### 3.2.4. Effect of Moxibustion Treatment on Trp-Kyn in the Hippocampus

**Figure 6:**
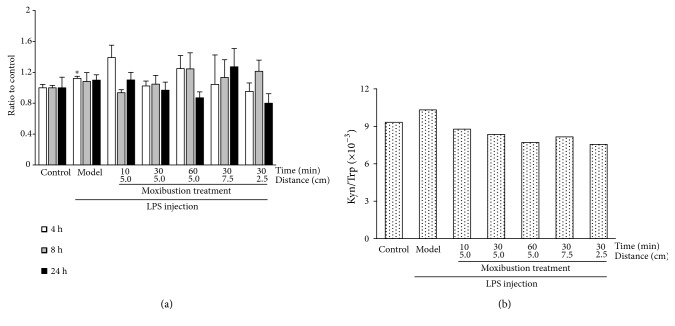
*Effects of moxibustion treatment on Trp metabolism via Kyn in hippocampus.* (a) Effect of moxibustion treatment on Kyn content in hippocampus, (b) Effect of moxibustion treatment on the ratio of hippocampus exposes of Kyn to Trp (Kyn/Trp). (*∗* P < 0.05: Compared with Control group).

An increased hippocampus Kyn content was observed in LPS-treated rats (Figure 6(a)). A decreased trend in hippocampus Kyn content was observed in some moxibustion-treated groups. The ratio of hippocampus exposure of Kyn to Trp (Kyn/Trp) was calculated to determine the metabolism capacity of the Trp-Kyn pathway [23, 24]. As shown in Figure 6(b), the Trp-Kyn metabolism capacity decreased with a prolonged duration of moxibustion treatment (8.78×10^−3^, 8.35×10^−3^ and 7.71×10^−3^). A nonlinear profile of moxibustion treatment temperature was also observed, in which the hippocampus Kyn/Trp exposure ratios were 8.16×10^−3^, 8.35×10^−3^, and 7.55×10^−3^.

#### 3.2.5. Interrelationships among the Behavioral Test Scores and Trp Metabolism Indexes

The Pearson correlation coefficients revealed that the behavioral test scores were positively related to the Trp level (P < 0.05) and negatively related to the Kyn/Trp ratio (P < 0.01) in the hippocampus (Table 4). These results indicate that, depressive symptoms had a strong correlation with the decrease in Trp level and the over-activation of the Trp-Kyn metabolism pathway. With the recovery of depressive symptoms after moxibustion treatment, an increased Trp level and reduced Kyn/Trp ratio were observed, proving that regulation of the Trp metabolism was an effective strategy in MDD therapy.

## 4. Discussion

Moxibustion treatment has long been used as a substitute therapy for depression. This study explored the underlying antidepressant mechanism and the sensitive moxibustion treatment factors in rats with LPS-induced depressant-like behavior. We determined that the benefit of moxibustion treatment on MDD is related to the combined effect of an elevated Trp transport and a shifted Trp metabolism in the hippocampus. Both duration and extent, two treatment strategy factors, are sensitive to the antidepressant effect of moxibustion treatment.

As the precursor of 5-HT, Trp depletion induces depressive-like behaviors, whereas Trp supplementation could improve depression by altering brain Trp availability [25–28]. In this study, moxibustion treatment increased the plasma levels of Trp, facilitating the brain uptake of Trp. Apart from the plasma Trp level, brain Trp availability is also determined by the transport capacity of brain blood vessels. As Trp competed the transporter with large neutral amino acids, the elevated plasma Trp level is also beneficial to increasing Trp transport capacity. Moreover, an increased brain blood Trp availability could be achieved by elevated brain blood perfusion under thermal stimulation in moxibustion treatment [29, 30]. Thus, the increased brain Trp uptake was considered an underlying anti-depression mechanism of moxibustion treatment.

Duration and temperature are two factors that influence the antidepressive efficacy of moxibustion treatment. To improve the brain uptake of Trp and thus achieve better efficacy, prolonging the treatment duration is an efficient approach. However, determining the optimal temperature for the brain uptake of Trp is more complex. In our study, the plasma Trp level was inversely related to the treatment extent. The weak-extent treatment group (7.5 cm in distance) exerted the highest plasma Trp level and Trp transport capacity (as shown in Ktp). Brain blood perfusion, another factor influencing the brain uptake of Trp, is positively related to the treatment extent. Despite having the lowest plasma Trp level, the Trp transport capacity in the group with a strong treatment extent (2.5 cm in distance) is superior to that in the group with an average treatment extent (5 cm in distance) with an average plasma Trp level. This study determined the complex influence of treatment temperature on Trp transport capacity. Further studies are required to explore the exact relationship between moxibustion treatment extent and Trp transport capacity.

Trp-5-HT and Trp-Kyn are two Trp metabolic pathways in the brain that are related to the disease progression of MDD. Expression of the key enzyme in the Trp-5-HT pathway, TPH2, is inhibited in depressive rat model [31], and brain 5-HT synthesis is decreased in depression patients [32–35]. The enhanced metabolism capacity of the Trp-5-HT pathway is a common feature of antidepressants [36] and has long been regarded as an important therapeutic strategy for MDD. Besides the 5-HT system, the aberrant over-activation of Trp-Kyn and the accumulation of Kyn metabolites have brought new insights into the progression of depression [37–39]. Therefore, elevating the Trp level and inhibiting the Trp-Kyn pathway activity are considered the most straightforward approaches [39]. Our data demonstrated that moxibustion treatment activated the Trp-5-HT pathway and suppressed the Trp-Kyn pathway. Such a shift in Trp metabolism pathways may be another underlying mechanism of moxibustion treatment.

In this study, we investigated changes in moxibustion efficacy on MDD under two altered treatment factors, duration and extent. The activation of Trp-5-HT metabolism is depended on both treatment duration and temperature. A prolonged treatment duration and an increased treatment extent could increase 5-HT generation. It is noteworthy that a shift in the Trp-5-HT pathway was more remarkable with changes in extent than changes in duration. This result agrees with behavioral tests that a more remarkable elevation was observed in the score of behavioral tests under an elevated extent rather than a prolonged duration. These results indicate that moxibustion efficacy is more determined by extent. These results indicate that moxibustion efficacy is determined more by extent. Generally, this study suggested that therapeutic extent plays a more important role in shifting the deleterious (Trp-Kyn) metabolism pathway of Trp to a beneficial (Trp-5-HT) one in the hippocampus. The extent of moxibustion affects antidepressive efficacy more, but further experiments are needed to optimize the extent. Prolonging the duration of moxibustion under the optimal extent is a highly feasible strategy for MDD treatment.

## 5. Conclusion

In this study, an increased brain uptake of Trp and a shifted Trp metabolism were identified as the underlying mechanisms of the beneficial effects of moxibustion treatment on depression-like behavior. The influence of therapeutic duration and extent on antidepressant effect was related to Trp transport and metabolism in the hippocampus. A prolonged therapeutic duration improved both Trp transport and metabolism to achieve a better antidepressant effect. As a more sensitive factor, therapeutic extent exerted a nonlinear influence on Trp transport. Further studies are needed to determine the optimal therapeutic extent for superior anti-depression moxibustion therapy.

## Figures and Tables

**Table 1:** Groups and treatment.

Group	Administration	Moxibustion treatment	
		Duration (min)	Extent (cm)
Control	Saline (i.p., 2.5 mL/kg)	-	-
Model	LPS (i.p., 0.5 mg/kg)	-	-
Mox1	LPS (i.p., 0.5 mg/kg)	10	5
Mox2	LPS (i.p., 0.5 mg/kg)	30	5
Mox3	LPS (i.p., 0.5 mg/kg)	60	5
Mox4	LPS (i.p., 0.5 mg/kg)	30	7.5
Mox5	LPS (i.p., 0.5 mg/kg)	30	2.5

**Table 2:** MRM transitions and parameters of analytes in plasma samples.

Analyte	Precursor ion (m/z)	Product ion (m/z)	Retention time (min)	Collision energy (eV)
Trp	205.8	146.1	3.53	18
Kyn	209.2	94.0	3.10	15
IS	166.2	148.1	3.58	11

**Table 3:** MRM transitions and parameters of analytes in hippocampus samples.

Analyte	Precursor ion (m/z)	Product ion (m/z)	Retention time (min)	Collision energy (eV)
Trp	309.4	263.1	7.03	18
5-HT	385.0	264.2	9.47	25
Kyn	417.3	122.1	8.07	25
IS	406.0	105.0	10.00	25

**Table 4:** Pearson correlation coefficients between score of behavioral tests and Trp metabolism indexes in hippocampus (N = 35^a^).

Indexes in Trp metabolism pathways	Pearson correlation coefficient	P value
Trp (*μ*g/g)	0.339	0.046
5-HT (ng/g)	0.287	0.095
Kyn (ng/g)	-0.271	0.116
Ktp (mL/g)	0.284	0.098
5-HT/Trp (×10-3)	0.208	0.230
Kyn/Trp (×10-3)	-0.538	0.001

^a^ Data at 24 h post LPS injection was used for analysis.

## Data Availability

All data used to support the findings of this study are included within the article.
